# Beyond the struggles: a scoping review on the transition to undergraduate clinical training

**DOI:** 10.1111/medu.13883

**Published:** 2019-04-23

**Authors:** Anique Atherley, Diana Dolmans, Wendy Hu, Iman Hegazi, Sonita Alexander, Pim W Teunissen

**Affiliations:** ^1^ School of Health Professions Education (SHE) Faculty of Health, Medicine and Life Sciences Maastricht University Maastricht the Netherlands; ^2^ School of Medicine University of Western Sydney Campbelltown New South Wales Australia; ^3^ The University of the West Indies Cave Hill Barbados; ^4^ Department of Obstetrics and Gynaecology VU University Medical Centre Amsterdam the Netherlands

## Abstract

**Context:**

The transition to clinical training within medical school is often seen as a struggle and students remain in distress despite numerous efforts to minimise threats. Efforts to change this may be misdirected if they are based on narrow conceptualisations of transitions. The authors conducted a scoping review to explore existing conceptual perspectives regarding the transition within medical school from pre‐clinical training to clinical training to suggest a research agenda and practical implications.

**Methods:**

Between October 2017 and February 2018 the authors searched PubMed, MEDLINE, ERIC, PsycINFO, Web of Science and CINAHL for English language literature with no date limits and retrieved 1582 articles; 46 were included in this review. Two reviewers independently screened articles and extracted data. Data were then charted, analysed and discussed with the research team.

**Results:**

The transition to clinical training was often described negatively as ‘difficult’, ‘a problem’ and ‘a struggle’. Our analysis found that researchers in medical education conducted studies on the transition to clinical training from three conceptual perspectives: educational; social, and developmental. Most research approached the transition to clinical training as a problem to be addressed from an educational perspective through transition to clerkship courses and curriculum innovations. Some research was conducted from a social perspective, focusing on building relationships. Regarding development, authors found a few articles highlighting opportunities for personal and professional development by nurturing transferrable learning strategies and reflection.

**Conclusions:**

This review provides an empirical base on which future research can be built to better understand and support medical students’ ability to navigate change. Finding new perspectives to approach the transition to clinical training could allow researchers to look beyond preparing students for struggles.

## Abstract 2

Atherley and colleagues review the literature on transitions from pre‐clinical to clinical training, offering conceptual perspective, practical guidance, and a research agenda.

## Introduction

Medical professionals are repeatedly exposed to changes in contexts and responsibilities from undergraduate to postgraduate training as they traverse the medical education continuum. Medical training steers students through pre‐clinical training, to clinical training, to being a newly practising doctor, to specialty training and ultimately to a consultant or specialist. This continuum is punctuated with prolonged, dynamic transition periods as a newcomer adjusts to a new environment.^1^, ^2^, ^3^, ^4^ Changing from primarily being in a ‘pre‐clinical’ classroom environment to mainly being in a ‘clinical’ patient‐focused environment is the first of many transition periods that medical trainees will encounter once they have begun medical training and are on a trajectory to being a doctor. This scoping review will focus on medical students transitioning from pre‐clinical to clinical training.

There is a large focus on increasing students’ preparedness and reducing the gap in knowledge and skills between pre‐clinical and clinical training.^5^, ^6^, ^7^, ^8^ A previous systematic review found that many aspects related to perceived preparedness during the transition from pre‐clinical to clinical training can be addressed in curricula.^9^ Such curricula changes include facilitating early patient contact^10^ and problem‐based learning.^11^ These interventions try to reduce the gap between pre‐clinical and clinical training, making the distinctions less apparent. However, despite these interventions, students still feel stressed and anxious,^8^, ^12^, ^13^ and as if they are ‘thrown into the deep end’^14^ when entering the clinical environment.

A previous scoping review by Yardley et al. provides a practical approach to transitions in medical education by describing the ‘dos, don'ts and don't knows’ of supporting trainees towards progressive independence during medical education. Yardley's review proposes moving beyond student perceptions when evaluating the impact of any transition,^3^ and further suggests that the lack of outcomes beyond students’^3^ perceptions could ‘have influenced the existent literature on the concept of transitions …’.^3^ This suggestion is likely to refer to the current framing, and thus focus, of transitions in the literature and it remains unclear what this constitutes. Although this review by Yardley et al. yielded practical suggestions for improving support during transitions, it did not explore the conceptualisations of transitions innate in the current understanding of transitions in medical education literature.^3^

In a recent commentary on the transition to postgraduate residency, O'Brien suggests that perhaps something is missing from the way transitions in medical education are framed and proposes a shift to framing this transition as a transformative process as opposed to a problem.^15^ The transition to clinical training within medical school might also benefit from a similar reframing of how it is currently perceived and conceptualised in medical education. Analysing existing conceptualisations could shed light on what current framings of the transition to undergraduate clinical training help us to understand, and what these conceptualisations prevent us from recognising. Such a synthesis could support future research on the transition to undergraduate clinical training. The authors conduct this review on the assumption that different interpretations of what transitions are and how they should be addressed are likely to influence research and practice, and we seek to set a baseline understanding. We therefore conducted a scoping review of the published literature on the transition to undergraduate clinical training to identify the conceptual perspectives taken when addressing the transition to undergraduate clinical training, as well as simultaneously identifying gaps in these perspectives.

## Methods

We conducted a scoping review following five stages as described by Levac et al.: (i) identifying the research questions; (ii) identifying the relevant studies; (iii) study selection; (iv) charting the data, and (v) collating, summarising and reporting results.^16^ Peters et al. describe that

‘… beyond preceding systematic reviews, scoping reviews are independently used to explore broad areas to identify gaps in the literature, clarify key concepts and report on the types of evidence that address and inform practice’.^17^

As such, scoping reviews are valuable for mapping the key concepts within a research area.^18^ Therefore, we conducted a scoping review in order to identify current conceptual perspectives taken in the literature regarding the transition to undergraduate clinical training and highlight gaps, in order to suggest a research agenda with some practical implications.

### Identifying the research question

This scoping review focused on the following research question: How have researchers approached the transition within medical school from pre‐clinical to clinical training and what are the gaps in these approaches? We did not seek to develop recommendations on how to improve current interventions relevant to this transition.

### Identifying relevant studies

We determined the search strategy through team discussion and consulting the university librarian. We searched PubMed, MEDLINE, ERIC, PsycINFO, Web of Science and CINAHL. A sample search strategy for PubMed is seen in Box 1 below, which yielded 52 articles. We used no date limits and included articles published online ahead of print. We conducted the initial search on 26 October 2017 and issued citation alerts until 28 February 2018.

Box 1 **Search strategy for PubMed**

transition[All Fields] AND ((((“clinical clerkship”[MeSH Terms] OR “clinical preceptorship”[All Fields]) OR “clinical clerkship”[MeSH Terms]) OR “clinical rotation”[All Fields]) OR “preceptorship”[MeSH Terms]) AND (((“medical student”[All Fields] OR “undergraduate”[All Fields]) OR “medical school”[All Fields]) OR “clinical student”[All Fields])

MeSH = Medical Subject Headings.

### Study selection

**Figure 1:**
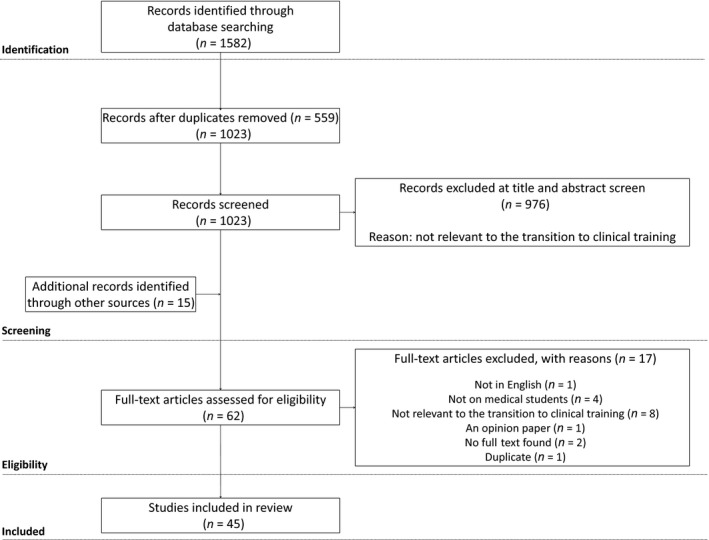
Flowchart of the search process and results for this scoping review

EndNote X8 (Clarivate Analytics, Philadelphia, PA, USA) was used to download the bibliographic details of studies yielded from the database searches and duplicates were deleted. Researchers AA and SA independently screened article titles and abstracts to determine eligibility for full‐text review against the inclusion criteria (Box 2). Any discrepancies were discussed until consensus. After this initial screening, AA and SA read full texts of articles to determine eligibility for inclusion. This scoping review included and excluded articles as per criteria in Box 2. Figure 1 shows a flowchart indicating this search and selection process. Our initial search was systematic in order to obtain a broad scope of the literature, with an aim to be inclusive in our review. Our scope of the literature yielded 1582 articles from six databases and 17 from reference screening and citation alerts. Following screening and full‐text review, 45 articles were included in this review.

Box 2 **Inclusion and exclusion criteria for this scoping review**

**Inclusion criteria**

The following articles were included in this scoping review:

1. Published in English
2. Focused on medical students, trainees or junior doctors (NOT dental, pharmacy, physiotherapy, nursing or other professions)
3. Discussed the transition to clinical training by:
  - describing or evaluating a support strategy that assists new undergraduate students in their transition to clinical training; and
  - describing students’ experiences during this transition as this was thought to yield insight into useful support strategies.
4. Was a review article including an exploration of the transition into clinical training
5. Was a theoretical article including an exploration of the transition into clinical training

**Exclusion criteria**

The following articles were excluded from this scoping review:

1. Perspective articles not substantiated by theory
2. Those without full text
3. Those exploring the transition to a single, specific undergraduate clerkship that was not the first clerkship, as students experiencing a second clerkship would have some previous full‐time experience within the clinical environment
4. Those evaluating transitions in patient care

### Charting the data

The first author (AA) developed a data charting form (see Appendix S1) to extract data, including author, publication year, journal, study aim, study design, theoretical framework, data collection methods, year of data collection, summary of key findings, description of a specific support strategy and relevant references. Another author (SA) reviewed full‐text articles against the data extraction to check for completeness and any discrepancies were discussed. The extraction process was iterative and was refined based on discussions between the first author (AA) and the rest of the research team (DD, WH, IH, SA and PWT).

### Collating, summarising and reporting results

The first author (AA) thematically analysed the data with SA and extracted data to answer the research question and meet the objectives of this scoping review. SA and AA discussed findings, which were then discussed with the rest of the research team (DD, WH, IH and PWT). atlas.ti Version 8.1.29.0 (ATLAS.ti scientific software development GmbH, Berlin, Germany) was used to help manage articles and any coding and synthesis of articles. This process was also iterative and bolstered by team discussions. Our team consists of a combination of clinicians, medical educators with doctorates and a current PhD candidate in medical education with previous research experience. Our syntheses of researchers’ perspectives were not explicitly described by researchers but instead represent our interpretations and reanalysis of existing research.

## Results

### Descriptive summary

Included studies were from the USA (*n* = 21), Europe (*n* = 11), UK (*n* = 8), Canada (*n* = 3) and Australia (*n* = 2). There were 12 qualitative studies,^10^, ^11^, ^14^, ^19^, ^20^, ^21^, ^22^, ^23^, ^24^, ^25^, ^26^, ^27^ 11 cross‐sectional surveys,^6^, ^7^, ^8^, ^13^, ^28^, ^29^, ^30^, ^31^, ^32^, ^33^, ^34^ eight descriptive case studies^5^, ^35^, ^36^, ^37^, ^38^, ^39^, ^40^, ^41^ (two of which were related, using the same participants and intervention),^36^, ^37^ seven longitudinal studies,^42^, ^43^, ^44^, ^45^, ^46^, ^47^, ^48^ three descriptive comparative studies,^12^, ^49^, ^50^ three review papers^1^, ^3^, ^9^ and one concept paper.^51^

### Conceptual perspectives

Our analysis found that researchers in medical education conducted studies on the transition to clinical training from three conceptual perspectives: educational, social and developmental. We do not suggest that these synthesised categories are mutually exclusive, but indicate the main focus of the articles in these categories. Allocation to a category is thus based on the implicit views of the transition from pre‐clinical to clinical training inherent in researchers’ discourse, interventions, choices of outcomes and suggestions. We will describe what we mean by these three perspectives, followed by details on the studies that were conducted from each perspective and what each one reveals regarding the transition to clinical training.

Box 3 shows a short description of our interpretations of these perspectives. Table 1 summarises these perspectives with regard to associated terminology, strategies used to approach the transition and annotations on a study that exemplifies each perspective.

Box 3 **Three conceptual perspectives used to approach the transition from pre‐clinical to clinical training**

**Educational perspective**

Implicit in this perspective was how researchers addressed students’ struggles by trying to narrow the gap between pre‐clinical and clinical training, often through courses and curriculum innovations to facilitate learning knowledge and skills.

**Social perspective**

Implicit in this perspective was how researchers addressed undergraduate students’ struggles when transitioning from pre‐clinical to clinical training by focusing on relationships and developing a nurturing learning environment^52^ between staff and students and between students themselves.

**Developmental perspective**

Implicit in this perspective was how researchers recognised that undergraduate students will always have challenges when transitioning from pre‐clinical to clinical training, but aimed to empower them by facilitating reflection and transferrable learning strategies.

**Table 1:** Perspectives taken on the transition from pre‐clinical to clinical training in the literature

	Frequently used associated transition terms	Focused on	Strategies to improve the transition	Summary of one example in the literature
Educational	Struggle Difficult Anxiety Stressful	Narrowing the gap between pre‐clinical and clinical training What students should know and be able to do	Transition to clerkship courses or orientations Problem‐based learning Early patient contact Targets knowledge, skills, logistics, assessment details	Chittenden et al.^12^ conducted a descriptive comparative study with 155 students who underwent a 7‐day, low‐stakes, high‐fidelity transition to clerkship course and compared perceived preparedness (in six skill sets), course satisfaction, performance in first clerkship and preceptor satisfaction to 147 controls who received a standard preparation for clerkships. Students felt more prepared for two of six skills, felt more confident and there was no difference in performance between groups.
Social	Struggle Difficult Anxiety Stressful	Cultural norms and fitting in Developing relationships with others	Introduction to staff to gain familiarity during orientations Targets students fitting in to the clinical team and peer and near‐peer relationships	Knobloch et al.^49^ described a mixed‐methods, descriptive case study with a historical cohort for comparison. They created a near‐peer‐led transition to clerkship seminar within a transition course. The seminar was 155 minutes and focused on integrating into teams, creating a study plan and a general question and answer session. In total 7 to 10 students were paired with two to three instructors. Students felt more prepared in the domains focused on immediately after the session and 6 months after.
Developmental	Challenging Opportunity Empowerment Trajectory	Personal and professional development Learning strategies and reflection	Portfolios Reflective interviews Targets learning strategies and promoting reflection	Pitkala and Mantyranta^42^ introduced a 1‐year portfolio in the first clinical year, which focused on learning diaries, narratives, logbooks, self‐evaluations and feedback from staff. Students reflected on feeling stressed and intimidated at the beginning but eventually enjoyed their student–doctor role. This strategy helped students to reflect on and recognise key experiences and supported professional development

#### Educational perspective

Researchers often portrayed the transition to clinical training negatively, describing it as difficult,^6^, ^39^, ^50^ stressful,^8^, ^12^, ^13^, ^19^, ^21^, ^23^, ^28^, ^35^, ^37^, ^47^, ^49^ anxiety generating^8^, ^12^, ^13^, ^32^, ^33^, ^34^, ^36^, ^37^ and a struggle.^7^, ^26^, ^27^, ^44^ Some studies started from a stance that there was a need to eliminate a variety of struggles, including students: feeling like they lacked the required knowledge and skills,^6^, ^11^, ^13^, ^19^, ^27^, ^34^ feeling unprepared,^11^, ^28^, ^30^, ^32^ feeling burdened by the demands of clinical training,^11^, ^19^, ^28^, ^42^ not meeting faculty members’ expectations,^9^, ^33^ and stressed by frequent changes in context.^27^

The tendency to address a gap in knowledge or skills led to researchers focusing on preparing students and reducing this gap. Researchers sought to ‘prepare students to excel as learners in clinical settings’^49^ by strengthening ‘students’ basic proficiency in oral patient presentations … basic skills in phlebotomy, arterial blood gases and suturing …’.^12^ Some research assumes that the aforementioned struggles will ‘stifle progress’.^28^, ^34^ This assumption is not supported by Van Hell et al.,^6^ who showed that perceived difficulty with the transition did not predict performance. Conceptualising the transition to clinical training from an educational perspective has influenced the creation of strategies aimed to ‘ease students’ transition from the pre‐clinical to clinical years’.^42^ These strategies include the development of specific transition to clerkship courses^5^, ^7^, ^8^, ^12^, ^35^, ^36^, ^37^, ^39^, ^40^, ^42^, ^49^, ^50^ and evaluating pre‐clinical curriculum innovations and their impact on students’ transition experiences.^6^, ^10^, ^11^, ^14^, ^22^, ^23^, ^24^, ^28^, ^29^, ^32^, ^33^, ^46^ Curriculum innovations in the literature include pre‐clinical problem‐based learning (PBL) curricula, 6, 11, 24, 28, 29, 32 creating space for early patient contact^10^, ^14^, ^33^ and development of longitudinal integrated clerkships (LIC) during clinical training.^22^

The aforementioned studies evaluated the transition to clinical training and relevant interventions with outcomes such as: student satisfaction with a particular strategy,^5^, ^12^, ^37^, ^39^, ^40^ measuring impact on anxiety^12^ and confidence^35^, ^42^ and calculating change in knowledge, skills or behaviour.^12^, ^35^, ^36^, ^37^, ^39^ These outcomes all reflect a focus on measuring the educational impact of a transition and related interventions. Some articles showed that transition‐to‐clerkship courses and curriculum innovations increased students’ perceived preparedness for clinical training,^12^, ^39^, ^49^ increased confidence,^9^, ^29^, ^35^, ^42^ reduced anxiety^12^ and motivated students.^24^ However, some students still struggled with socialisation.^10^, ^23^

Most literature on the transition to clinical training was from an educational perspective, which sees the transition as a struggle due to the knowledge and skill deficits that students have. Consequently, interventions were designed to address these deficits with pre‐clinical courses and inductions. As a result, studies focus on outcomes such as student satisfaction and perceived preparedness, with some showing increased preparedness and student satisfaction. We recognised a second perspective with a shared concern for students’ struggles when entering clinical training but a different approach to managing their difficulties.

#### Social perspective

Again, researchers described the transition to clinical training in negative terms and highlighted a need to eliminate a variety of struggles, including students: trying to fit in with insiders,^32^, ^46^, ^48^ feeling intimidated by others,^32^, ^34^ being unsure of their role,^9^, ^27^ and learning the cultural norms of the clinical environment.^27^ Studies suggest that students were ‘unfamiliar with learning within the workplace and uncertain about how to navigate and engage within teams and culture they have not come to understand’.^43^ As a result, students often placed a significant focus on fitting in with the clinical team as opposed to learning.^46^

Addressing students’ integration led to a focus on peer–peer and peer–team relationships. Researchers thought it was important to make explicit ‘the hidden knowledge that students need to become effective team members’^25^ and to create strategies that would ‘describe the roles and expectations … routines and logistics’ relevant to the clinical environment.^12^ It therefore was important to facilitate meaningful interactions and relationship building with others.^25^, ^43^, ^48^ Examples of these social strategies included initiating peer groups,^25^ near‐peer teaching sessions,^48^ facilitating students sharing experiences,^43^ and creating peer learning communities.^45^ Additionally, to promote student–staff relationships in the clinical environment, some researchers reported on the use of multidisciplinary approaches to educational inductions and including residents in these orientations.^12^, ^39^ Regarding measuring outcomes, increased social support was likely to reduce stress^19^ and, in one study, increased students’ perceptions of preparedness as it related to integrating into the clinical team.^48^

Literature on the transition to clinical training conducted from a social perspective still sees the transition as a struggle as a result of students trying to fit into the new environment and not being familiar with existing cultural norms and how to build relationships. The approach was therefore to develop activities that familiarise students with others (professionals and peers) in the clinical environment. As a result, studies focused on outcomes related to fitting into the clinical team.

This social perspective alone does not seem to recognise the importance of students’ self‐awareness and reflection to minimise the impact that negative role modelling could have on novice students. We recognised a third conceptualisation of the transition to clinical training that differs from the first two in that it doesn't problematise students’ struggles.

#### Developmental perspective

Unique to this perspective, researchers reported the transition to clinical training as challenging.^1^, ^5^, ^7^, ^9^, ^36^, ^37^, ^38^, ^43^, ^44^, ^50^ This terminology differs to that of the other two perspectives and could be considered a positive cognitive appraisal by researchers highlighting the potential for students’ growth.^52^, ^53^ Research within this perspective allows the discussion to shift away from a stance of minimising particular struggles that students experience during the transition to clinical training and towards recognising the need for students to be able to cope with change.

Conceptualising the transition to clinical training from a developmental perspective has led to the creation of strategies aimed at empowerment. By contrast with researchers’ focus on problems from educational and social perspectives, when taking a developmental perspective, researchers mentioned the desire ‘to empower third‐year undergraduate medical students to recognize learning opportunities in their clinical placements and to proactively use them to develop their understanding and practice’.^43^ Empowering students promotes personal and professional development by optimising learning strategies and encouraging reflection.^38^, ^41^, ^43^, ^44^, ^50^

The literature provides some examples of how researchers approached development during the transition to clinical training. The transition to clinical training requires that students adapt their learning strategies to learn in a self‐directed way but this does not automatically develop.^44^ Additionally, the clinical environment could have a negative impact on self‐regulated learning skills^44^ by increasing extrinsic goal orientation when decreasing metacognitive self‐regulation.^44^ Optimising clinical learning strategies is therefore important for new clinical students. Additionally, reflection in the form of portfolios^41^ and narratives towards the end of pre‐clinical training^38^ facilitated students recognising key experiences,^38^, ^39^, ^41^ promoted professional development^41^ and reframed their experiences towards becoming the type of doctor they wanted to be.^38^

There was comparatively less research carried out from a social and developmental perspective. However, researchers are increasingly recognising a need to explore the opportunities that the transition to clinical training can provide.^1^, ^30^, ^44^

## Discussion

This scoping review demonstrates that the transition from pre‐clinical to clinical training is conceptualised from three perspectives: educational, social and developmental. Most research was undertaken from an educational perspective as compared to social and developmental approaches. Here we explore what these three perspectives say and do not say about the transition to clinical training. Then, we will briefly describe practical implications of our findings and suggest a research agenda.

Having an educational perspective primarily focuses researchers on reducing the gap between pre‐clinical and clinical training and produces a desire to increase students’ knowledge and skills so that students experience a smaller gap between these training stages. In this light, a ‘good’ transition from an educational perspective, is likely to be one where students feel prepared, have all the knowledge and skills required to start clinical training and do not feel overwhelmed by the amount of learning to cover. However, it is not enough for students to feel prepared^3^, ^54^ nor should we expect that we can adequately prepare them for the dynamics of a new environment, which itself is unstable. This is not to suggest that educational preparation is not important; however, this is not the sole factor. Kilminster described transitions in postgraduate training to be critically intensive learning periods.^54^ Even though the concept was in a postgraduate setting, using this conceptualisation within medical school is likely to be useful for minimising the focus on preparedness and, instead, promoting the transition as a dynamic period in which students learn and establish relationships.

A social perspective on the transition to clinical training fills the gaps in taking a solely educational perspective by reducing the expectation that students need to be knowledgeable and skilled, thus allowing a focus on building relationships with staff, peers or near‐peers. Cultivating a nurturing medical environment where students are not intimidated can encourage team building and student engagement.^55^ Most research has focused on targeting students and very rarely explicitly focuses on training staff to help students to integrate within clinical teams.^39^, ^50^ Students still experience academic bullying and negative role modelling during the transition to clinical training^56^, ^57^, so it is likely that the clerkship culture and environment need to adapt with newly arriving clinical students to create a new community functioning *with* newcomers. This would require the newcomers to be integrated into the daily work of the environment, which could promote learning and motivation and could even add value to the community.^58^ A ‘good’ transition from a social perspective is likely to be one where students integrate into the community of practice of the clerkship environment and gain legitimate access to learning opportunities through participation. This perspective, however, might be overlooking the usefulness of social support being used alongside self‐reflection to help students decipher which behaviours should be imitated.^59^

A developmental perspective fills gaps in both the educational and social perspectives on the transition to clinical training by promoting student reflection on learning and integration experiences. Taking this developmental perspective empowers students and provides them with the tools for ownership of their learning and transition experience through reflection and optimising transferrable learning skills. Transitions provide opportunities to offer proactive and reactive support and learn coping skills, which are imperative for future learning as medical trainees.^60^ Amongst interns (recent medical school graduates), Liu et al.^61^ found that using self‐directed learning as a coping strategy led to motivation and learning as compared to emotion‐based strategies such as avoidance. This highlights that sometimes ‘struggles’ could serve as motivation for learning.^61^ Proactive support strategies, as opposed to reactive strategies occurring when difficulties already exist, are likely to be particularly useful to help trainees learn to deal with stressful change, which is highly likely in their career.^60^ Promoting developmental skills such as reflection, self‐regulated learning skills and other skills such as resilience, could help students adapt to change.^2^, ^3^, ^60^ A ‘good’ transition from a developmental perspective might start from a stance where researchers understand that the transition is challenging but can be harnessed to provide motivation for learning.

We have illustrated how a particular perspective could influence approaches, outcomes and perceptions of what is a ‘good transition’. In practice, it could be beneficial to combine elements of educational, social and developmental perspectives. This combination could result in students’ education prior to the transition period being appropriate, so they can integrate socially and utilise developmental competencies of reflection and resilience.

### A new research agenda

Our findings highlight areas that may be overlooked by research that adopts single conceptualisations of the transition to clinical training. There is comparatively less research from social and developmental perspectives. We suggest that research from a social or developmental perspective could still be useful in order to inform combined social and developmental approaches to the transition to clinical training. Research from a social perspective could ask: How do relationships with others aid students’ transition experiences and learning when entering the clinical environment? From a developmental perspective, it could be useful to understand: Which procedures for reflection during the transition would be most beneficial to students’ experiences? Lastly from a combined perspective one might explore: How could reflection influence students’ relationships during the transition to clinical training? Methodologically, longitudinal research might be informative from a developmental perspective to allow researchers to understand the process of professional and personal growth during challenging transition periods such as entering clinical training.

Existing research rarely showed researchers explicitly reflecting on their potential biases and assumptions when conducting research on the transition to clinical training. Future research could benefit from researchers critically considering from which perspective they are approaching the transition to clinical training and the impact this might have on their methodology, findings and interpretations.

Combining researcher reflexivity with underpinning empirical research with theory could be powerful next steps in researching the transition to clinical training. For example, stressful experiences can trigger transformative learning, which Mezirow suggests requires experiences, critical reflection and dialogue with others in order to transform individuals’ existing perspectives of themselves, their beliefs and their behaviours.^62^ Future researchers could conceptualise the transition to clinical training as a transformational experience, thereby combining educational, social and developmental perspectives. O'Brien posits that medical educators should recognise that the transition is ‘an adaptable learning process’^15^ and speaks to the potential for transformative learning^62^ during the postgraduate transition to residency; this could be transferrable to medical school experiences,^3^ as well as priming postgraduates for future careers. Future research could, therefore, explore what a transformative transition would look like and how this could be evaluated. Focusing on a new conceptualisation of the transition to clinical training – being a transformative experience – could allow new outcomes for research in this area to be considered and researchers might decrypt what a successful transition might look like, what outcomes are important, how to measure them, and effective ways to support students’ transitions.

This scoping review also provides evidence that the scales currently tip towards the fact that researchers consider the transition to clinical training to be a threat. Lazarus and Folkman suggest that feelings of threat and challenge may occur simultaneously as students transition into clinical training and, although related, challenge and threat may not be on the same continuum, but instead are separate constructs.^53^ There is much research on the transition as a threat but less on transition as a developmental challenge.

### Limitations

Additional databases may have yielded more articles. Non‐English articles might have been overlooked. Additionally, we made the decision to exclude grey literature. The focus of our scoping review was on the scholarly conceptualisations of the phenomenon of transitioning to clinical training. Our results may be helpful for reflecting on the grey literature as well and we suspect there is a strong focus on the educational perspective, for example, through educational innovation reports. The first author (AENA) has had an interest in transitions for over 5 years, which might have led to the development of preconceived notions surrounding the transition to clinical training and influenced interpretations of data. However, discussions with the research team are likely to have reduced this bias.

## Conclusions

This scoping review provides insight into perspectives found in the literature on the transition from pre‐clinical to clinical training within medical school. This transition is primarily seen as a maladaptive struggle, with many researchers addressing the transition from an educational perspective by focusing on increasing preparedness with relevant knowledge and skills. However, the challenge associated with the transition to clinical training can be motivating and be an important, critically intensive learning period for new clinical students. Future research on the transition to clinical training from social and developmental perspectives (individual and combined) is likely to stimulate opportunities to advance students’ adaptations to the clinical environment.

## Contributors

AA, DD and PWT conceptualised the study. AA, DD, WH, IH and PWT were involved in the design of the study. AA and SA were responsible for data collection and analysis. AA, DD, WH, IH, SA and PWT were involved in the interpretation of data. AA produced the first draft of the paper but all authors (AA, DD, WH, IH, SA and PWT) contributed to iterative drafting and refinement of the manuscript. All authors (AA, DD, WH, IH, SA and PWT) approved the final version of the manuscript for submission.

## Funding

AA is supported by a scholarship through the Western Sydney University as part of a joint PhD collaboration between Western Sydney University and Maastricht University.

## Conflicts of interest

none.

## Ethical approval

not applicable.

## Disclaimers

none.

## Supporting information


**Appendix S1.** Data charting form.
