# Supplementary material for: Beyond the struggles: a scoping review on the transition to undergraduate clinical training
Source: Med Educ. 2019 Apr 23;53(6):559–70. doi: 10.1111/medu.13883 (PMC6593677; doi:10.1111/medu.13883)
Supplement: Supplementary file 1 — Appendix S1. Data charting form. [file MEDU-53-559-s001.docx]

**Appendix**

**Data charting form**

| 1. Author(s): 2. Year of publication: 3. Journal: 4. Study aim: 5. Study design: 6. Theoretical framework used for methods or to interpret results: 7. Data collection methods: 8. Year of data collection 9. Summary of key findings: 10. Description of a specific support strategy: 11. References that could be included: |
| --- |
